# TREM2 promotes the formation of a tumor-supportive microenvironment in hepatocellular carcinoma

**DOI:** 10.1186/s13046-025-03287-w

**Published:** 2025-01-21

**Authors:** Hanrui Guo, Meiling Wang, Caiya Ni, Chun Yang, Chunxue Fu, Xiaoman Zhang, Xueling Chen, Xiangwei Wu, Jun Hou, Lianghai Wang

**Affiliations:** 1 Key Laboratory of Xinjiang Endemic and Ethnic Diseases, Shihezi University School of Medicine, Shihezi, Xinjiang China; 2 NHC Key Laboratory of Prevention and Treatment of Central Asia High Incidence Diseases, First Affiliated Hospital, Shihezi University School of Medicine, Shihezi, Xinjiang China; 3 Department of Clinical Laboratory, Jinan Maternity and Child Care Hospital, Affiliated to Shandong First Medical University, Jinan, Shandong China; 4 Department of Pathology, Jinan Maternity and Child Care Hospital, Affiliated to Shandong First Medical University, Jinan, Shandong China; 5 Department of Pathology, Affiliated Tianfu Hospital of Southwest Medical University (Meishan Tianfu New Area People’s Hospital), Meishan, Sichuan China

**Keywords:** TREM2, HCC, CD8, Glycolysis, PKM2

## Abstract

**Background:**

Triggering receptor expressed on myeloid cells 2 (TREM2), a surface receptor predominantly expressed on myeloid cells, is a major hub gene in pathology-induced immune signaling. However, its function in hepatocellular carcinoma (HCC) remains controversial. This study aimed to evaluate the role of TREM2 in the tumor microenvironment in the context of HCC progression.

**Methods:**

HCC was experimentally induced in wild-type (WT) and *Trem2*-deficient (*Trem2*^−/−^) mice, and clinical sample analysis and in vitro studies on macrophages were conducted. HCC cells were treated with conditioned medium from WT or *Trem2*^−/−^ macrophages, and their malignant phenotypes and underlying mechanisms were analyzed.

**Results:**

*TREM2* deficiency reduced liver tumor burden in orthotopic and subcutaneous HCC models by altering CD8^+^ T cell infiltration. *Trem2*-deficient macrophages presented increased chemokine secretion. TGF-β1 was found to be positively correlated with TREM2 expression in HCC, and TGF-β blockade reversed TREM2 induction. On the other hand, TREM2^+^ macrophages were found to be associated with glycolysis and PKM2 expression in HCC cells; this association may be related to the secretion of IL-1β, which enhances the malignant phenotypes of HCC cells.

**Conclusions:**

These results reveal that TREM2^+^ macrophages play a driving role in HCC progression by suppressing CD8^+^ T cell infiltration and promoting tumor cell glycolysis, providing a new therapeutic target for HCC.

**Supplementary Information:**

The online version contains supplementary material available at 10.1186/s13046-025-03287-w.

## Introduction

Hepatocellular carcinoma (HCC) is a significant cause of cancer-related death worldwide, and its incidence rate continues to rise [1]. Unfortunately, the prognosis of HCC is generally poor. Recently, interest in modulating the immune response as a novel approach for treating tumors has increased [2, 3]. However, the objective remission rate for advanced HCC is currently only approximately 20% [4]. Therefore, increasing the effectiveness of immunotherapy in treating HCC is crucial.

Triggering receptor expressed on myeloid cells 2 (TREM2) is a transmembrane glycoprotein that is expressed mainly on the surface of myeloid cells [5]. TREM2 plays a crucial role in inflammation-related diseases by mechanisms such as sensing inflammation during tissue injury, which can impact nerve regeneration, atherosclerosis, and fatty liver [5–7]. In microglia and macrophages, TREM2 can act as an anti-inflammatory receptor that negatively regulates Toll-like receptor (TLR)-mediated inflammatory responses [5, 8]. Furthermore, when Kupffer cells were isolated from *Trem2*^*−/−*^ mice and stimulated with TLR4, the proinflammatory response was enhanced [9]. Therefore, TREM2, which acts as an immunosuppressive factor, may induce a chronic immunosuppressive state in tumor-associated macrophages.

Increasing evidence suggests that TREM2 plays an essential role in various tumors. Studies have shown that high expression of TREM2 is associated with poor prognosis in patients with gastric cancer [10], glioma [11], and renal cell carcinoma [12]. However, the role of TREM2 in hepatocarcinogenesis remains controversial. Some groups have proposed that TREM2 acts as an oncogene in HCC, with elevated expression in tumors [13]. Our previous study demonstrated that TREM2 is expressed predominantly by a macrophage subpopulation enriched in HCC tissues [14]. In contrast, other studies identified TREM2 as a tumor suppressor in HCC that acts via the PI3K/Akt/β-catenin pathway [15]. *Trem2* deficiency exacerbates liver injury and inflammatory responses, increasing the number of liver tumors in chemical-induced injury models, suggesting that TREM2 plays a protective role in hepatocarcinogenesis [16]. Therefore, further investigations are warranted to explore the role of TREM2 in HCC and its impact on the tumor microenvironment.

In this study, we investigated the role of TREM2^+^ macrophages in various mouse HCC models. We employed transcriptome sequencing analysis and a cytokine array to assess differences in the levels of cytokines secreted by TREM2^+^ macrophages. Additionally, we elucidated the regulatory effect of TGF-β1 signaling on TREM2^+^ macrophages. We further investigated the role of TREM2^+^ macrophages in promoting the malignant phenotypes of HCC cells. These findings contribute to a better understanding of the multifaceted roles of TREM2 in HCC and provide insights into personalized treatment approaches for HCC patients with high TREM2 expression.

## Materials and methods

### Animal models

To establish a chemically induced HCC model, we injected diethylnitrosamine (DEN) (25 mg/kg) intraperitoneally once into two-week-old male C57BL/6 mice. Additionally, we injected 10% CCl_4_ (0.5 mL/kg) intraperitoneally starting at 4 weeks of age once a week for 20 weeks. The mice were euthanized, and tissues were collected at 24 weeks of age.

For the orthotopic HCC model, we implanted 3 × 10^6^ Hepa 1–6 cells (1:1 mixed Matrigel, Corning) into the livers of 4-week-old male wild-type (WT) and *Trem2*^*−/−*^ C57BL/6 mice (NM-KO-190402, Shanghai Model Organisms Center, Inc.) and euthanized them for tissue harvesting three weeks later. Trem2 gain-of-function experiments were conducted via intravenous tail vein injection of *Trem2*-expressing adeno-associated viruses (AAV-Trem2; AAV8-F4/80-Trem2, Hanheng Biotechnology) or AAV-vehicle (1.2 × 10^11^ vector genomes) seven days before the orthotopic injection of Hepa 1–6 cells.

To establish a subcutaneous HCC model, we injected 3 × 10^6^ Hepa 1–6 cells (1:1 mixed Matrigel, Corning) into the ventral blood supply-rich area of four-week-old male mice. Tumor growth was continuously observed, and tumor dimensions were recorded for three weeks. Then, the mice were euthanized for tissue harvesting. CD8^+^ T cell depletion was conducted by intraperitoneal injection of *InVivo*MAb anti-mouse CD8α (200 µg/mouse; BE0061, BioXCell) every three days for a total of six times, starting three days after tumor cell inoculation. The tumor volume was estimated using the following formula: (length * width^2^)/2.

The grouping of all the animals was randomized. The experiments were approved by the Animal Experimental Ethical Inspection of the First Affiliated Hospital, Shihezi University.

### Human samples

Formalin-fixed paraffin-embedded samples from 109 patients with HCC who did not receive radiotherapy or chemotherapy and underwent surgical resection between 2011 and 2019 were collected at the First Affiliated Hospital of Shihezi University. The study was approved by the Science and Technology Ethical Committee of the First Affiliated Hospital, Shihezi University.

### Immunohistochemistry (IHC)

Sections of paraformaldehyde-fixed, paraffin-embedded mouse liver samples (4 μm thick) and human HCC tissue microarrays were subjected to antigen retrieval using a pressure cooker. Endogenous peroxidase activity was inhibited via the addition of 3% hydrogen peroxide. Nonspecific binding was blocked using 10% goat serum. The sections were subsequently incubated with antibodies against mouse Trem2 (1:300, ab86491, Abcam), CD8 (1:800, #98941, Cell Signaling Technology), CD4 (1:800, BS6982, Bioworld Technology), FOXP3 (1:800, ab215206, Abcam), cleaved caspase-3 (1:800, #9661, Cell Signaling Technology), TGF-β1 (1:800, ab215715, Abcam), PKM2 (1:1600, #4053, Cell Signaling Technology), and human TREM2 (1:1000, #91068, Cell Signaling Technology) at 4 °C overnight. The sections were then incubated with secondary antibody (ZSGB-BIO) and DAB substrate and counterstained with hematoxylin. Images were acquired via a KF-PRO-005 Digital Slide Scanner (KFBIO). The final staining scores of CXCL10 and PKM2 were calculated by multiplying the score for the percentage of positive cells (range, 0–4; 0, negative or ≤ 5%; 1, 6–25%; 2, 26–50%; 3, 51–75%; and 4, 76–100%) by the intensity score (range, 0–3; 0, negative; 1, weak; 2, moderate; and 3, strong) [17]. The final staining score of TREM2 was the product of the intensity score (range, 0–3; 0, negative; 1, weak; 2, moderate; and 3, strong) and the percentage of positive cells (range, 0–100) as previously described [18]. The staining score of TGF-β1 was determined as the percentage of positive cells per field.

### Cell culture

Bone marrow cells were collected by washing the femurs and tibias of WT and *Trem2*^*−/−*^ mice with PBS. After the red blood cells were lysed, the cell suspension was filtered through a 70-µm sieve. The cells were cultured in DMEM supplemented with murine recombinant M-CSF (40 ng/mL; 315-02, PeproTech) at 37 °C in a humidified 5% CO_2_ atmosphere for seven days, differentiating into mouse bone marrow-derived macrophages (BMDMs). Fresh complete DMEM was used for culture for 24 h, and the supernatant of the medium was collected as conditioned medium (CM).

Authenticated human monocytic THP-1 and mouse Hepa 1–6 cells without mycoplasma contamination were obtained from the Cell Bank, Type Culture Collection, Chinese Academy of Sciences. THP-1 cells were maintained in RPMI-1640 medium (Gibco) supplemented with 10% fetal bovine serum (FBSST-01033, Cyagen), 5 µM 2-mercaptoethanol (21985023, Gibco), and 1% penicillin-streptomycin. THP-1 cells were exposed to phorbol 12-myristate-13-acetate (PMA; CS0001, Multi Sciences) at 50 ng/mL for 48 h to differentiate into macrophage-like cells. Hepa 1–6 cells were maintained in complete DMEM (D6429, Sigma-Aldrich) supplemented with 10% fetal bovine serum and 1% penicillin-streptomycin.

### Cytokine assay

The cell culture supernatant of the BMDMs was collected and centrifuged to remove particulates. Next, cytokines and chemokines in the cell culture supernatants were profiled via the Mouse Cytokine Array, Panel A (ARY006, R&D Systems), according to the manufacturer’s instructions. Images were captured using a chemiluminescence imaging system (Clinx Science Instruments).

### Real-time PCR

Total RNA was extracted using the E.Z.N.A. Total RNA Kit (R6834-01, Omega Bio-Tek) and reverse transcribed into cDNA via the HiFiScript cDNA Synthesis Kit (CW2569M, CWBIO) following the manufacturer’s instructions. Gene expression analyses were conducted via TB Green Premix Ex Taq II (Takara) on a CFX96 Touch Real-Time PCR Detection System (Bio-Rad Laboratories). *ACTB* served as the internal control. The primers used are listed in Supplementary Table 1.

### Western blotting

Protein lysates were prepared from cells using RIPA lysis buffer (R0010, Solarbio) containing protease and phosphatase inhibitors (P1260, Solarbio). The protein concentration was determined via a BCA protein assay (CW0014S, CWBIO). Proteins were separated by 10% or 12% SDS‒polyacrylamide gel electrophoresis and transferred onto Immobilon-FL PVDF membranes (IPLH00010, Millipore). The membranes were incubated overnight at 4 °C with primary antibodies against human TREM2 (1:1000, #91068, Cell Signaling Technology), mouse Trem2 (1:1000, AF1729, R&D Systems), PKM2 (1:1000, #4053, Cell Signaling Technology), and β-actin (1:1000, #TA-09, ZSGB-BIO). The membranes were subsequently incubated with secondary antibodies for 2 h at room temperature. The bands were detected using ECL Western Blotting Substrate (BL520A, Biosharp).

### Cell proliferation assay

Hepa 1–6 cells were seeded in 96-well plates at a density of 3000 cells per well, with BMDM CM mixed with complete medium (1:2). Cell proliferation was assessed at 0, 24, 48, and 72 h using the Enhanced Cell Counting Kit-8 (BL1055B, Biosharp) according to the manufacturer’s instructions.

### Data collection and processing

The Cancer Genome Atlas Liver Hepatocellular Carcinoma (TCGA-LIHC) dataset was utilized for gene expression correlation analysis, survival analysis, and enrichment analysis, and the results were then validated with the GSE14520 dataset obtained from the Gene Expression Omnibus (GEO). The list of genes encoding secreted proteins was compiled from the Human Protein Atlas (https://www.proteinatlas.org/). Molecules capable of regulating the glycolytic pathway were identified via a literature search (Supplementary Table 2).

### Gene set enrichment analysis

Gene set enrichment analysis for all the hallmark gene sets associated with *TREM2* expression in TCGA-LIHC and GSE14520 datasets was conducted, and the data were visualized; both analysis and data visualization were performed using the “GSVA” package in R [19].

### Identification of differentially expressed genes

We used the “DEseq2” package in R to identify differentially expressed genes [20]. Differential expression was analyzed between WT BMDMs and *Trem2*^−/−^ BMDMs based on log2-transformed gene expression levels. Genes with |log FC| ≥ 0.585 and *P* < 0.05 were considered significantly differentially expressed.

### ELISA

The cell culture supernatant was diluted 30 times, and the IL-1β concentration was assessed using the Mouse IL-1β ELISA Kit (EK201B, MULTISCIENCES) according to the manufacturer’s instructions. Finally, the OD values were measured via an enzyme-linked immunosorbent assay. The final OD value = OD_450_ value-OD_630_ value.

### Glucose consumption and lactic acid production

A total of 5 × 10^5^ cells were cultured in 6-well plates for 24 h and then cultured for another 24 h with BMDM CM mixed with complete medium (1:2) with or without raleukin (50 ng/mL; HY-108841, MedChemExpress). The medium was collected, and the changes in the glucose consumption and lactate production capacity of the cells were detected via the Glucose Assays Kit-WST (G264, DojinDo) and Lactic Acid (LA) Content Assay Kit (BC2235, Solarbio) [21, 22].

### Statistical analysis

Numerical data are presented as the means ± SDs and were analyzed via GraphPad Prism and R software. The Kaplan‒Meier method was used to analyze patient survival, and the log-rank test was performed for statistical analysis. A t test or ANOVA was used to analyze the significance of differences between groups. Correlations between genes were assessed according to Spearman correlation coefficients. Differences were considered statistically significant at a *P* value < 0.05.

## Results

### TREM2 deficiency reduces liver tumor burden

**Fig. 1:**
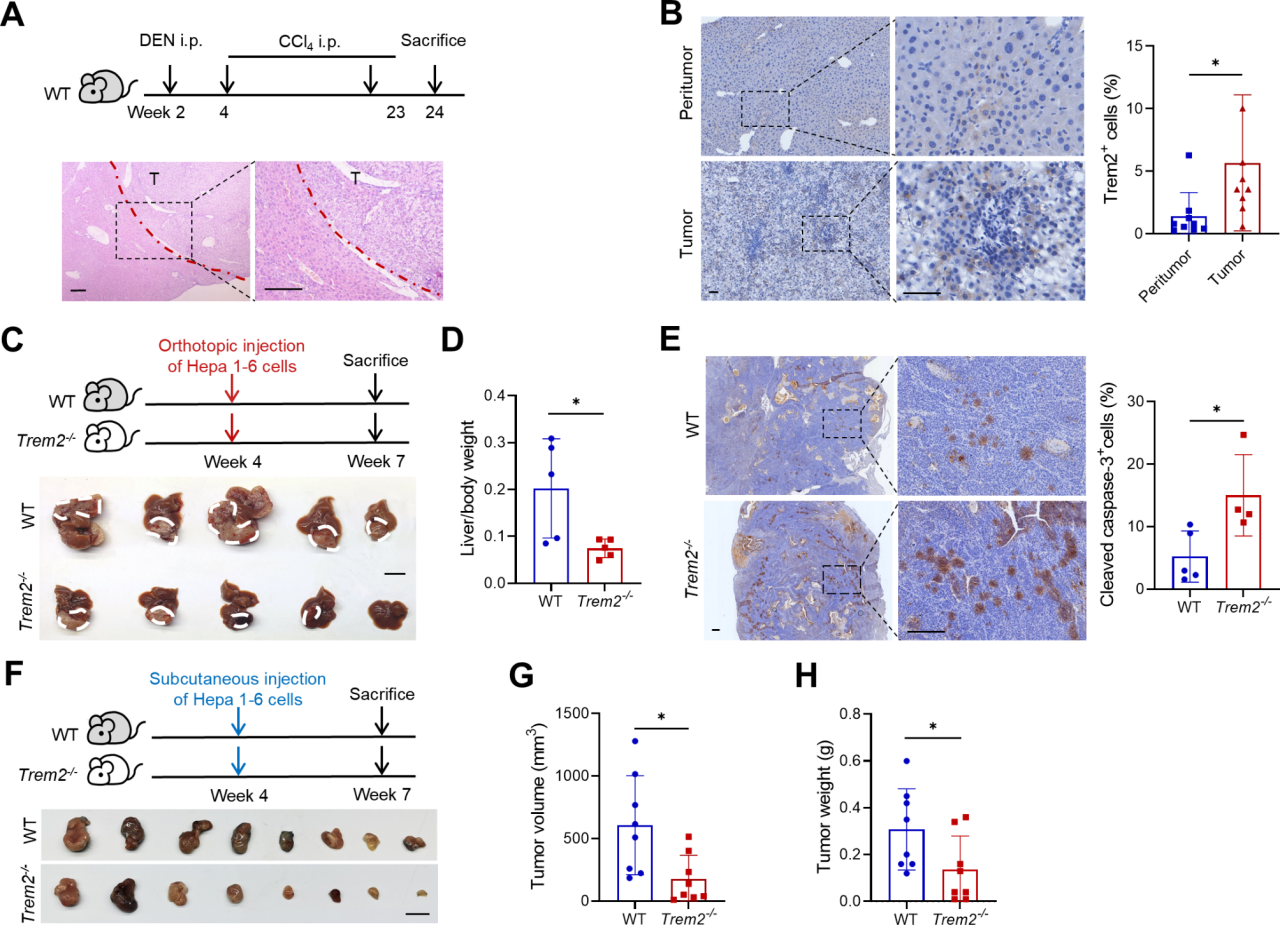
TREM2 deficiency reduces liver tumor burden. (**A**) C57BL/6 mice were intraperitoneally (i.p.) injected with DEN once (25 mg/kg) starting at two weeks of age, followed by 20 weekly injections of CCl_4_ (0.5 mL/kg); the mice were subsequently sacrificed at 24 weeks. Liver tissue was collected for H&E staining. Scale bar, 200 μm. (**B**) Representative graphs of IHC staining for TREM2 in chemically induced murine HCC and paraneoplastic tissues. Scale bar, 50 μm. (**C**) Hepa 1–6 cells were injected into the livers of wild-type (WT) and *Trem2* knockout (*Trem2*^*−/−*^) mice at four weeks of age to establish an orthotopic model of HCC. The mice were sacrificed and analyzed three weeks after injection (*n* = 5). Scale bar, 1 cm. (**D**) Statistical analyses of the mouse liver/body weight ratio. (**E**) Representative images of IHC staining for cleaved caspase-3 in liver tumors and corresponding statistics showing the percentage of positively stained cells. Scale bar, 200 μm. (**F**) The subcutaneous HCC model established with WT and *Trem2*^*−/−*^mice (*n* = 8). Scale bar, 1 cm. (**G**, **H**) Statistical analyses of tumor volume (**G**) and tumor weight (**H**) in the mice. *P < 0.05, ***P < 0.001

To investigate the role of TREM2 in HCC, we initially induced liver tumors in mice by administering DEN and CCl_4_. The success of model construction was confirmed through hematoxylin and eosin (H&E) staining (Fig. 1A). The results from the IHC analysis revealed that in mouse liver tumors induced by DEN and CCl_4_, there was a significant increase in the number of TREM2^+^ cells compared with that in peritumoral tissues (Fig. 1B). To further explore the involvement of TREM2^+^ cells in the progression of HCC, an orthotopic HCC mouse model was established by injecting Hepa 1–6 cells intrahepatically into wild-type (WT) and *Trem2*-knockout (*Trem2*^*−/−*^) mice. Deletion of *Trem2* suppressed orthotopic HCC growth, as characterized by decreases in the number of tumors and the liver/body weight ratio (Fig. 1C and D). The proportion of cleaved caspase-3^+^ cells in tumors from *Trem2*^*−/−*^ mice was significantly greater than that in tumors from WT mice (Fig. 1E), suggesting enhanced tumor cell killing after *Trem2* deficiency. Similarly, *Trem2* knockout inhibited tumor growth in the subcutaneous HCC model, as indicated by decreases in tumor volume and weight (Fig. 1F‒H). These findings indicate that TREM2 plays a tumor-promoting role during HCC development.

### TREM2 deficiency attenuates tumor growth by modifying CD8+ T cells

**Fig. 2:**
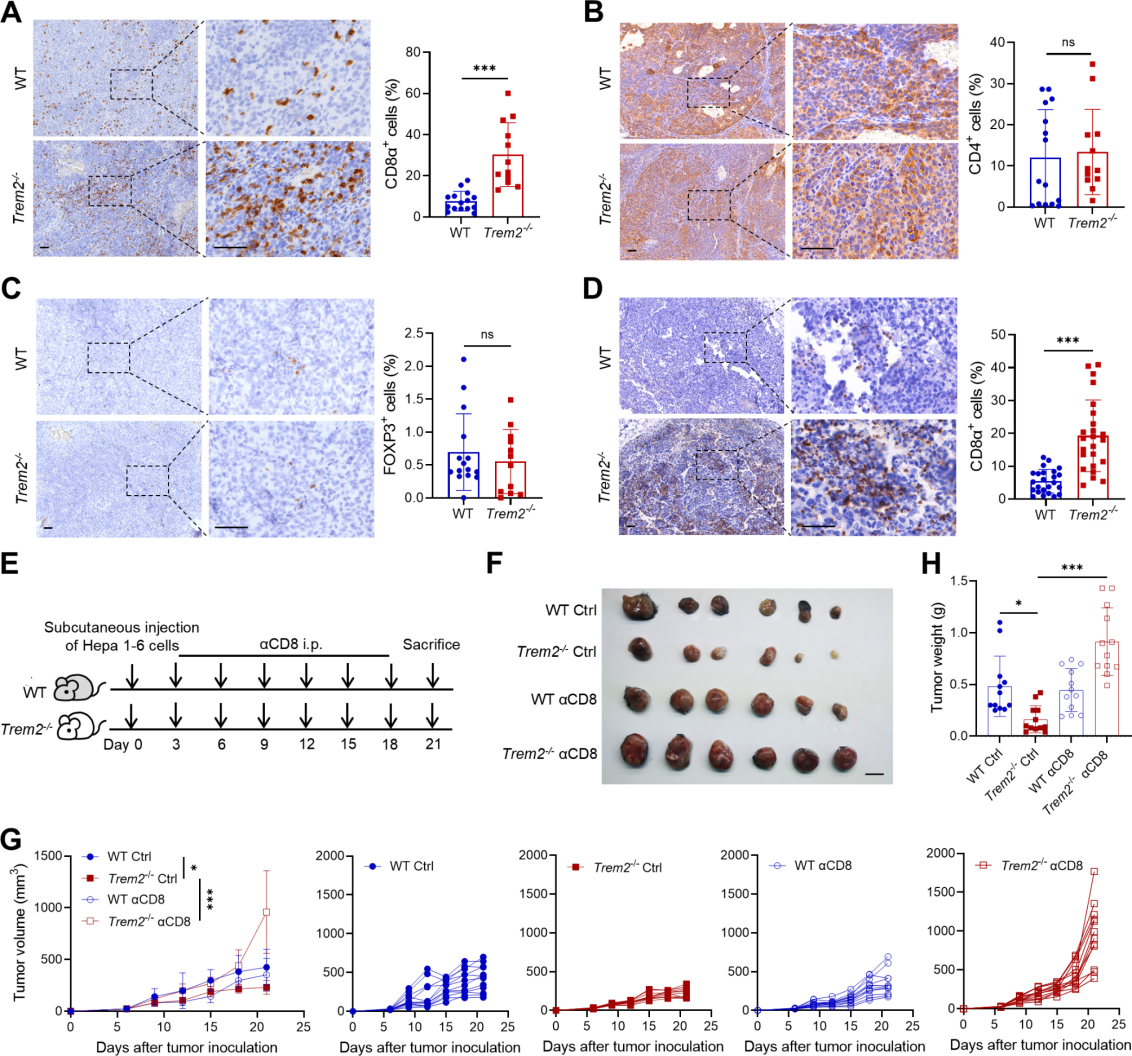
TREM2 deficiency attenuates tumor growth by modifying CD8^+^T cells. (**A**‒**C**) IHC staining and quantification of CD8α (**A**), CD4 (**B**), and FOXP3 (**C**) expression in orthotopic liver tumors from WT and *Trem2* knockout mice (*n* = 15 tumor areas from five WT mice and *n* = 12 tumor areas from four *Trem2*^*−/−*^ mice). Scale bar, 50 μm. (D) IHC staining and quantification of CD8α in mouse subcutaneous HCC samples (*n* = 24 tumor areas from eight mice). Scale bar, 50 μm. (**E**) Subcutaneous tumor models of HCC in WT and *Trem2*^*−/−*^ mice were intraperitoneally (i.p.) injected with a CD8 depletion antibody every three days starting on day 3. (**F**) Tumors were harvested on day 21, and images were obtained (*n* = 6). Scale bar, 1 cm. (**G**) Tumor growth curves of the WT and *Trem2*^*−/−*^ groups and single mice after depletion of CD8^+^ T cells. Pool of two experiments (*n* = 12). (**H**) Statistical analysis of the tumor weight. ns, not significant; *P < 0.05, ***P < 0.001

Next, we conducted immunohistochemistry (IHC) analysis on tumors obtained from the orthotopic HCC model to investigate the impact of TREM2 on the composition of major immune cell populations associated with tumor growth. The staining results revealed that CD8α^+^ cells were extensively distributed in the tumors from the *Trem2*^*−/−*^ mice, whereas very few CD8α^+^ cells were observed in the WT mice (Fig. 2A). Nevertheless, no significant difference in CD4^+^ or FOXP3^+^ cells was detected between WT and *Trem2*^*−/−*^ mice (Fig. 2B and C). Similarly, the subcutaneous HCC model also presented increased CD8^+^ T cells in tumors from *Trem2*^*−/−*^ mice (Fig. 2D). To directly investigate the impact of CD8^+^ T cells on the pro-oncogenic effect of TREM2, we depleted CD8^+^ T cells with an antibody in the subcutaneous HCC model (Fig. 2E). We observed that the inhibition of tumor growth induced by *Trem2* knockout was abolished upon CD8^+^ T cell depletion, as indicated by the restored tumor volume and weight (Fig. 2F‒H). These findings confirm that CD8^+^ T cells play a predominant role in mediating the effects of TREM2 on HCC progression.

To corroborate the above findings, we performed gain-of-function experiments involving AAV-mediated overexpression of *Trem2* in liver macrophages in a mouse orthotopic HCC model (Supplementary Fig. 1A). The liver/body weight ratios were greater in the AAV-Trem2 group than in the AAV-vehicle group (Supplementary Fig. 1B and C). IHC analysis revealed a significant decrease in the proportion of CD8a^+^ cells in tumors from *Trem2*-overexpressing mice (Supplementary Fig. 1D).

To further explore the impact of TREM2^+^ macrophages on CD8^+^ T cells in the tumor microenvironment, we intrahepatically injected Hepa 1–6 cells in combination with WT or *Trem2*^*−/−*^ BMDMs into WT mice and then harvested liver tumors for single-cell sequencing at the experimental endpoint. Major cell types and myeloid cell subclusters were identified (Supplementary Fig. 2A and B). In line with a recent report that CXCL9 and SPP1 indicate tumor-associated macrophage polarity in human cancers [23], *Cxcl9* expression was significantly higher in Trem2^−^ macrophages, whereas *Spp1* expression was significantly upregulated in Trem2^+^ macrophages (Supplementary Fig. 2C). Moreover, differential gene expression analysis revealed that the expression of *Gzmb* was significantly greater in proliferating CD8^+^ T cells and memory CD8^+^ T cells in tumor samples derived from the *Trem2*^*−/−*^ BMDM group than in those derived from the WT BMDM group (Supplementary Fig. 2D and E). These results suggest that TREM2 may promote the formation of a microenvironment with fewer CD8^+^ T cells and increase the tumor burden in HCC.

### *Trem2* -deficient macrophages show enhanced chemokine secretion

**Fig. 3:**
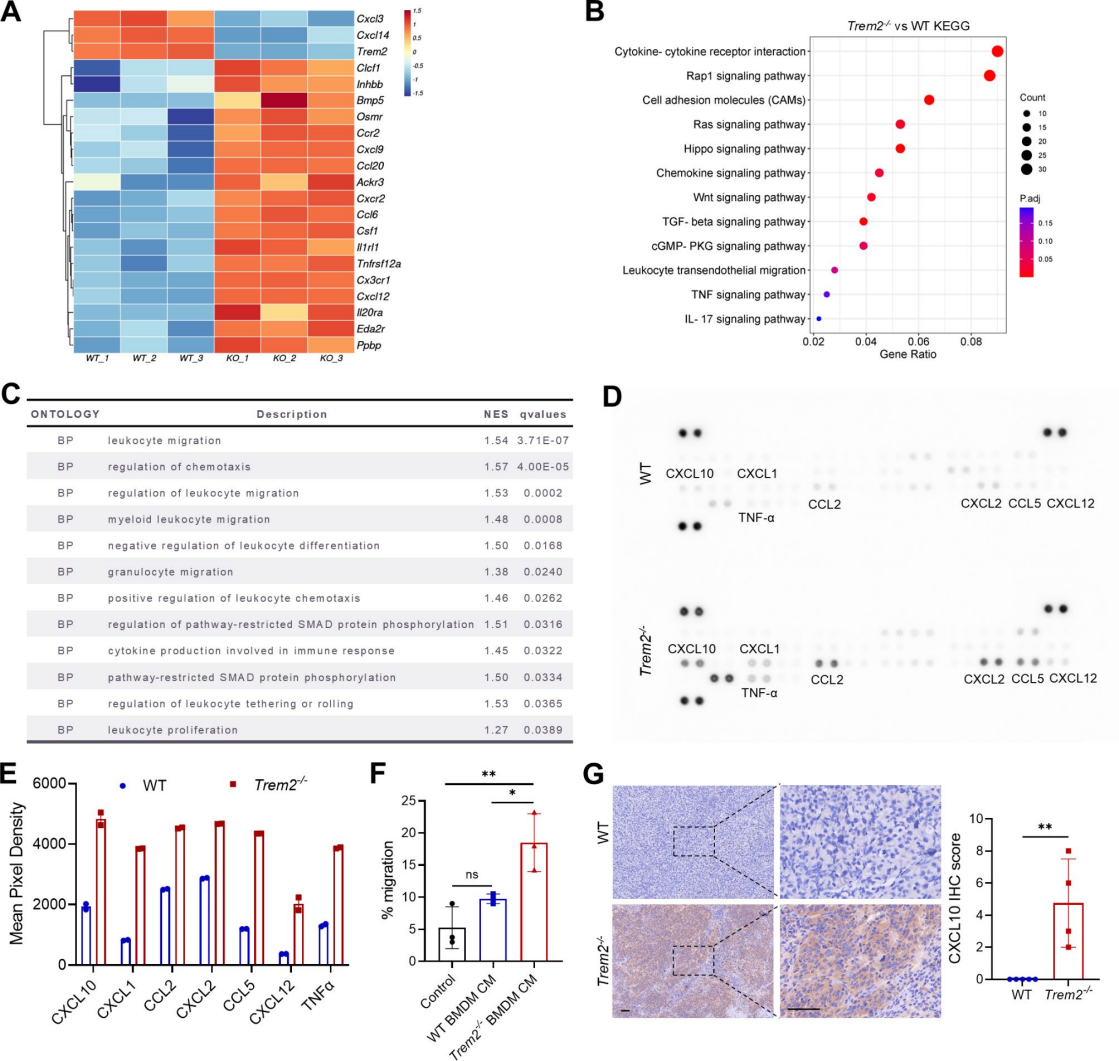
*Trem2* -deficient macrophages show increased chemokine secretion. (**A**) Heatmap visualization of RNA sequencing data of BMDMs from WT and *Trem2*^*−/−*^ mice. (**B**) Enrichment analysis of KEGG pathways. (**C**) Gene set enrichment analysis of Gene Ontology biological processes (GO-BPs). (**D**) Cytokine microarray assay of WT and *Trem2*^−/−^ BMDM culture supernatants. (**E**) Quantification plots of differential cytokine levels. (**F**) Mouse splenocytes were treated with the culture supernatant of BMDMs from WT and *Trem2*^−/−^ mice for 12 h. Cell migration was assessed via a Transwell assay, and the number of migrating cells was counted under a microscope. (**G**) IHC of CXCL10 was performed on mouse orthotopic HCC tissues. Scale bar, 50 μm. *P* < 0.05, ** *P* < 0.01

To investigate the mechanism by which TREM2 promotes tumor immunosuppression, we performed RNA sequencing of BMDMs derived from WT and *Trem2*^*−/−*^ mice. The results revealed elevated expression of cytokines and chemokines in *Trem2* knockout BMDMs (Fig. 3A). We also observed significant enrichment of pathways including cytokine-cytokine receptor interaction, chemokine signaling pathway, leukocyte migration, and chemotaxis (Fig. 3B and C). Additionally, we collected cell culture supernatants from WT and *Trem2*^*−/−*^ BMDMs for a mouse cytokine array (Fig. 3D). *Trem2*-deficient BMDMs secreted increased levels of chemokines and cytokines, including CXCL10, CCL5, and TNF-α (Fig. 3E). Transwell assays revealed that CM from *Trem2*^*−/−*^ BMDMs was more effective at inducing lymphocyte migration than CM from WT BMDMs (Fig. 3F). IHC analysis of liver tumors from the orthotopic HCC model further confirmed increased levels of CXCL10 in the *Trem2*-deficient group (Fig. 3G).

### TREM2 expression is correlated with TGF-β1 expression in HCC

**Fig. 4:**
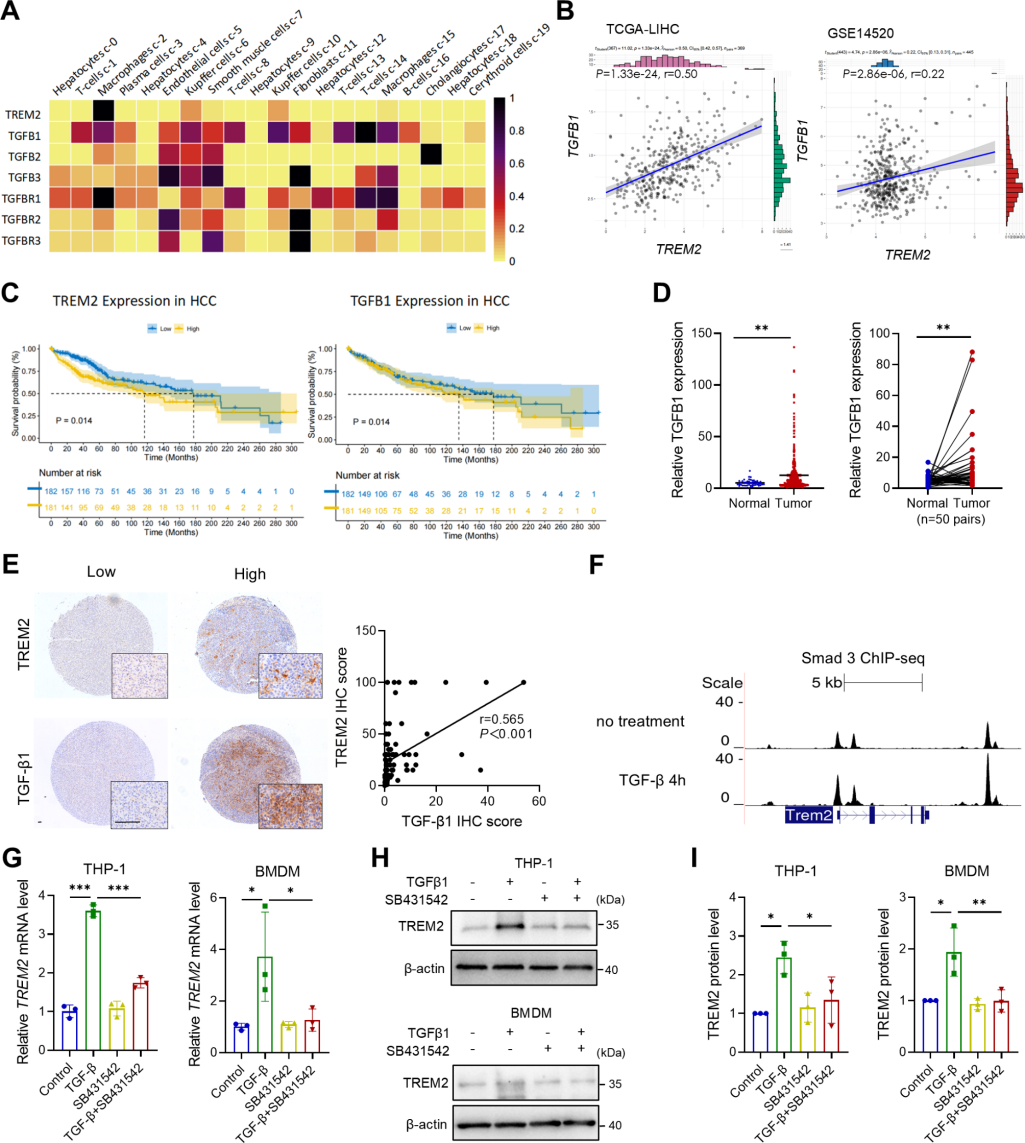
TREM2 expression is positively correlated with TGF-β1 in HCC. (**A**) Heatmap of the expression of *TREM2* and the indicated genes in the TGF-β family in single cell types of the human liver (GSE115469) derived from the Human Protein Atlas. (**B**) Correlations between *TGFB1* and *TREM2* in TCGA-LIHC and GSE14520 cohorts. (**C**) Kaplan‒Meier curves of the overall survival of patients with high/low expression of *TREM2* and *TGFB1* in TCGA-LIHC dataset. (**D**) *TGFB1* expression in normal (*n* = 50) and tumor tissues (*n* = 371) from TCGA-LIHC dataset. (**E**) IHC staining and correlation of TREM2 and TGF-β1 levels in a human HCC tissue microarray. Scale bar, 100 μm. (**F**) Visualization of Smad3 ChIP-seq (GSE72964) peaks around the *Trem2* locus in untreated and TGF-β-treated BMDMs. (**G**) *TREM2* expression in human THP-1 cells and mouse BMDMs in the presence of TGF-β (50 ng/mL) and SB431542 (10 nM), a TGF-β receptor kinase inhibitor. (**H**, **I**) Representative blots and statistical analysis of TREM2 levels in THP-1 cells and BMDMs in the presence of TGF-β and SB431542 by western blotting. β-actin was used as the internal control. *P < 0.05, ** *P* < 0.01, ***P < 0.001

A recent study revealed that TGF-β signaling is involved in the development of nonalcoholic steatohepatitis-associated macrophages (NAMs), which express molecular markers, including TREM2, in the liver [24]. Analysis of the scRNA-seq dataset revealed that *TGFB1*, *TGFB2*, and *TGFB3* are expressed by diverse cell types in the human liver, whereas *TGFBR1*, but not *TGFBR2* or *TGFBR3*, is highly expressed in TREM2^+^ macrophages (Fig. 4A). There was a significant positive correlation between the expression levels of *TREM2* and *TGFB1* in TCGA-LIHC and GSE14520 datasets (Fig. 4B, Supplementary Fig. 3A and B), indicating that TGF-β signaling may play a role in inducing TREM2^+^ macrophages in HCC. Moreover, high *TREM2* and *TGFB1* expression was associated with poorer overall survival in TCGA-LIHC cohort (Fig. 4C). Additionally, the expression of *TGFB1* was significantly elevated in tumor samples compared with that in normal liver tissues (Fig. 4D). IHC analysis of our cohort of HCC patients revealed that the TGF-β1 protein level was positively correlated with the TREM2 protein level in HCC (Fig. 4E). Analysis of the ChIP-sequencing data revealed stronger Smad3 binding peaks around the *Trem2* locus in BMDMs following TGF-β1 treatment (Fig. 4F). To explore the potential induction of TREM2^+^ macrophages by TGF-β signaling, human THP-1 cells and mouse BMDMs were treated with TGF-β1 and SB431542, a TGF-β receptor kinase inhibitor. Following treatment with TGF-β1, significant increases in the mRNA and protein levels of TREM2 were observed in THP-1 cells and BMDMs, whereas TGF-β blockade reversed the increase in TREM2 levels induced by TGF-β1 (Fig. 4G‒I). Compared with vehicle control treatment, SB431542-mediated inhibition of TGF-β in the orthotopic HCC model resulted in smaller lesions (Supplementary Fig. 3C). Moreover, Tumor IMmune Estimation Resource (TIMER) analysis of TCGA-LIHC dataset revealed a negative correlation between the expression of *TREM2* and *TGFB1* and the infiltration levels of naive CD8^+^ T cells (Supplementary Fig. 3D). These findings suggest that TGF-β1 can promote TREM2^+^ macrophage induction.

### TREM2^+^ macrophages are associated with glycolysis and PKM2 expression in HCC cells

**Fig. 5:**
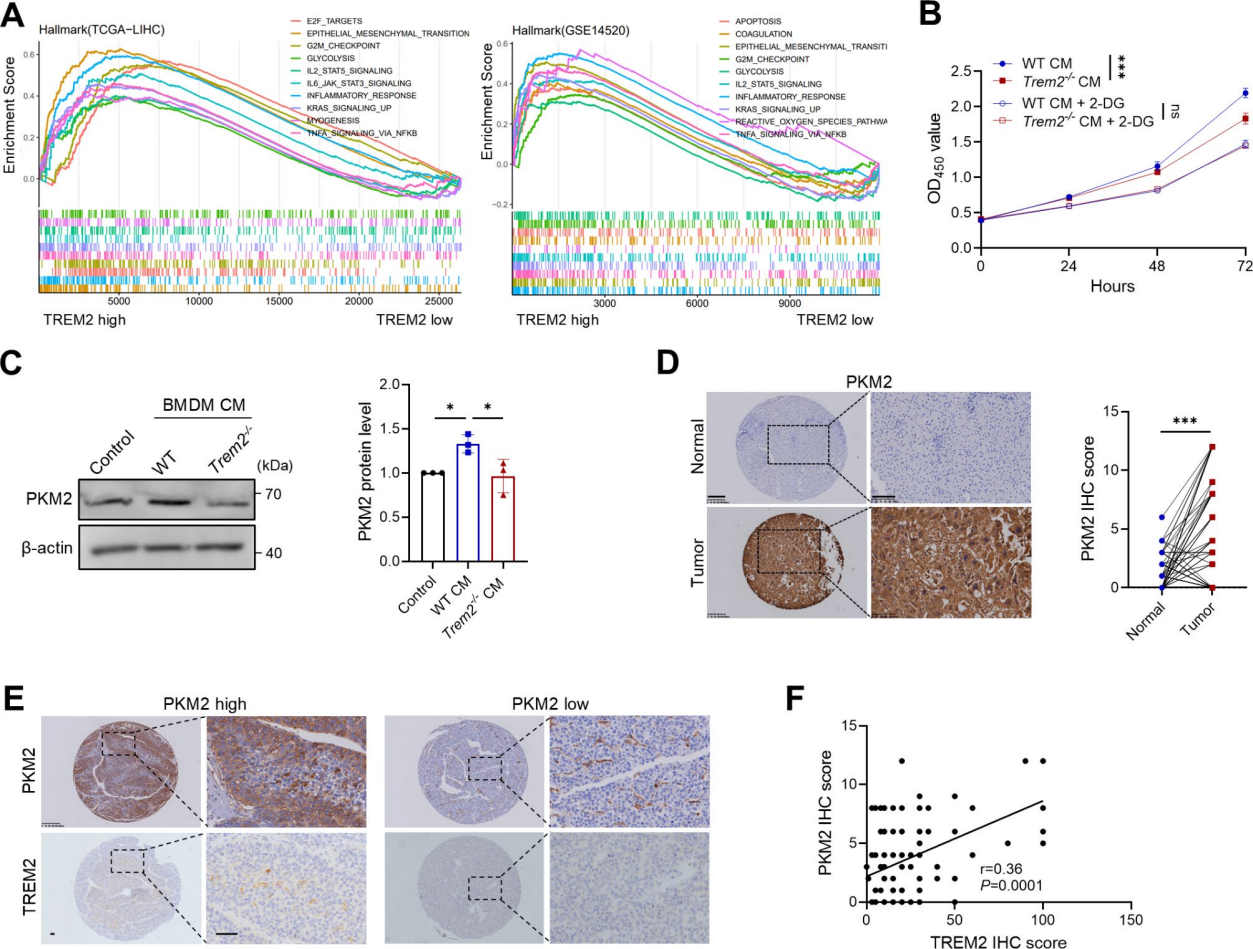
TREM2^+^macrophages are associated with glycolysis and PKM2 expression in HCC cells. (**A**) Hallmark gene set enrichment in TCGA-LIHC (left) and GSE14520 (right) datasets. (**B**) Changes in the proliferation of Hepa 1–6 cells treated with conditioned medium (CM) form WT and *Trem2*^*−/−*^ BMDMs in the presence or absence of 2-DG (2 mM). (**C**) The levels of PKM2 protein in Hepa 1–6 cells treated with the indicated CM types were assessed via western blotting. (**D**) Representative images (left) and statistical analysis of PKM2 staining in adjacent nontumor liver tissues and HCC samples (*n* = 84 pairs). (**E**) Representative images of TREM2 staining in human HCC samples with different PKM2 levels. Scale bar, 50 μm. (**F**) Correlation between TREM2 and PKM2 levels in HCC samples (*n* = 109). ns, not significant; *P < 0.05, ** *P* < 0.01, ***P < 0.001

Since studies have shown that glycolysis is significantly activated in HCC [25], we wondered whether TREM2^+^ macrophages are involved in this process. Gene set enrichment analysis of TCGA-LIHC and GSE14520 datasets revealed that the glycolytic pathway was enriched in the high *TREM2* expression group (Fig. 5A). We collected CM from WT and *Trem2*^*−/−*^ BMDMs and treated Hepa 1–6 cells with the CM. We found that the proliferation of Hepa 1–6 cells in the WT group was significantly greater than that in the *Trem2*^*−/−*^ group (Fig. 5B). However, when 2-deoxyglucose (2-DG) was used to inhibit glycolytic function [26], the differences in the proliferation of Hepa 1–6 cells between the WT and *Trem2*^*−/−*^ groups were eliminated (Fig. 5B), indicating that the TREM2^+^macrophage-promoted malignant phenotypes of HCC are related to glycolysis.

Next, we explored how TREM2^+^ macrophages regulate glycolysis in HCC cells. We identified 12 genes that were highly positively correlated with *TREM2* expression and the frequency of *TREM2*^*+*^ macrophages in TCGA-LIHC dataset, including three genes related to glycolysis, namely, *PKM*, *G6PD*, and *ALDOA* (Supplementary Fig. 4A‒C). Survival analysis revealed that high expression of these genes was associated with poor prognosis in TCGA-LIHC and GSE14520 datasets (Supplementary Fig. 4D‒F). To verify whether TREM2^+^ macrophages regulate the expression of PKM, G6PD, and ALDOA in tumor cells, we detected their levels in Hepa 1–6 cells treated with CM from WT or *Trem2*^*−/−*^ BMDMs. The results revealed a significant difference in *Pkm* mRNA expression between the WT and *Trem2*^*−/−*^ groups but not for *G6pd* and *Aldoa* (Supplementary Fig. 4G). PKM2, an isoform of PKM, can form dimers in the nucleus and participate in the regulation of energy metabolism [27]. Further analysis revealed that CM from WT but not *Trem2*^*−/−*^ BMDMs triggered PKM2 expression at the protein level in Hepa 1–6 cells (Fig. 5C). IHC staining of the tissue microarray revealed that PKM2 levels were significantly higher in human HCC samples than in adjacent normal tissues (Fig. 5D). Correlation analysis with TREM2 staining revealed a positive association between the levels of TREM2 and PKM2 in HCC samples (Fig. 5E and F). These results demonstrated that TREM2^+^ macrophages can induce PKM2 expression in HCC cells.

### TREM2^+^ macrophages induce PKM2 expression in HCC cells by IL-1β secretion

**Fig. 6:**
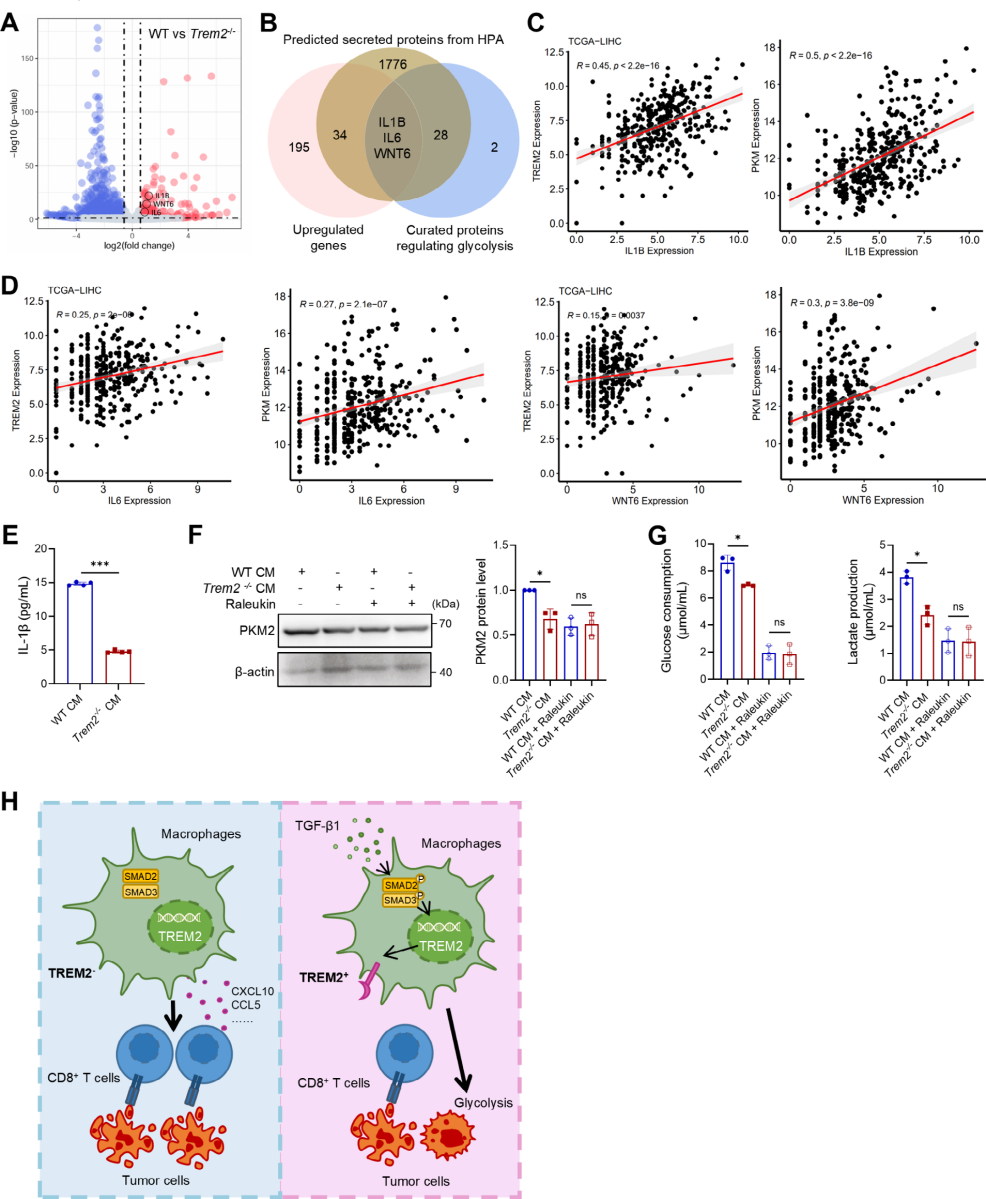
Secretion of IL-1β by TREM2^+^macrophages increases PKM2 expression and promotes glycolysis in HCC. (**A**) Differentially expressed genes between WT and *Trem2*^−/−^ BMDMs according to RNA-sequencing analysis. (**B**) The intersection of genes upregulated in WT BMDMs compared with *Trem2*^−/−^ BMDMs, predicted secreted proteins obtained from the Human Protein Atlas (HPA), and curated proteins regulating glycolysis. (**C**) Correlations between *IL1B* and *TREM2* expression in TCGA-LIHC (left) and GSE14520 (right) datasets. (**D**) Correlations of *IL6* and *WNT6* expression with *TREM2* expression in TCGA-LIHC and GSE14520 datasets. (**E**) Secretion of IL-1β in the CM of the indicated BMDMs. (**F**) PKM2 levels in Hepa 1–6 cells treated with the indicated CM with or without the IL-1R antagonist raleukin (50 ng/mL). (**G**) Glucose consumption and lactate production in Hepa 1–6 cells treated with the indicated CM types in the presence or absence of raleukin. (**H**) Schematic diagram of the tumor-promoting role of TREM2^+^ macrophages in HCC. ns, not significant; *P < 0.05, ***P < 0.001

To determine which component secreted by TREM2^+^ macrophages causes the malignant progression of HCC, we conducted an intersection of the genes whose expression was upregulated in WT BMDMs compared with *Trem2*^*−/−*^ BMDMs according to RNA sequencing (Fig. 6A), the predicted secreted proteins obtained from the HPA database, and the curated proteins regulating glycolysis, which revealed three overlapping candidate genes, namely, *IL1B*, *IL6*, and *WNT6* (Fig. 6B). Correlation analysis revealed that only *IL1B* expression was strongly associated (*R* > 0.3) with *TREM2* expression in TCGA-LIHC and GSE14520 datasets (Fig. 6C and D). ELISA confirmed that the concentration of secreted IL-1β was significantly greater in the culture supernatant from WT BMDMs than in that from *Trem2*^*−/−*^ BMDMs (Fig. 6E). The difference in PKM2 levels in Hepa 1–6 cells treated with CM from the WT and *Trem2*^*−/−*^ BMDM groups was eliminated by blocking IL-1 signaling with the human IL-1R antagonist raleukin (Fig. 6F). Similarly, the differences in glucose consumption and lactic acid production between the two groups were abolished by raleukin treatment (Fig. 6G). These results demonstrate that the secretion of IL-1β by TREM2^+^ macrophages may upregulate PKM2 expression and promote glycolysis in HCC cells.

## Discussion

In recent years, immune checkpoint inhibitors have become commonly used in HCC treatment. Therapies such as atezolizumab plus bevacizumab and tremelimumab plus durvalumab have been approved as standard first-line treatments [28]. However, these treatments may have limited efficacy in most patients with advanced HCC [29]. To achieve precision medicine in HCC in the future, it is necessary to explore more biomarkers and therapeutic targets [30]. In this study, we observed increased expression of TREM2 in tumors in a chemically induced mouse model of primary HCC. In our comparison of THP-1-derived macrophages with a panel of commonly used HCC cells through western blotting, we observed that TREM2 was highly expressed in macrophages but expressed at minimal levels in HCC cells (Supplementary Fig. 5A). This finding aligns with prior scRNA-seq analyses and dual-color immunofluorescence staining of human HCC samples [14, 16]. To further investigate the impact of TREM2 on HCC development, we conducted experiments using orthotopic and subcutaneous transplantation tumor models. Our results revealed that *Trem2* knockout reduced the tumor load, decreased the liver/body weight ratio, and promoted tumor cell death. In human HCC, patients with higher TREM2 IHC scores had a greater recurrence ratio compared to those with lower TREM2 IHC scores (55.9% vs. 32.4%; Supplementary Fig. 5B). The level of *TREM2* expression also increased in patients with advanced disease grade and stage (Supplementary Fig. 5C). These findings suggest that TREM2 plays a role in promoting tumor development in HCC. Our findings are consistent with those of previous studies reporting a tumor-promoting role of TREM2 in other types of tumors, including sarcoma, colorectal cancer, and breast tumors [31]. The combination of therapies targeting TREM2 with immune checkpoint blockade using anti-PD-1 or anti-PD-L1 antibodies may enhance therapeutic effects in various cancers [31–33].

In contrast to the findings of this study regarding the role of TREM2 in HCC progression, previous studies have suggested that TREM2 might act as a protective factor during hepatocarcinogenesis. It negatively regulates hepatic inflammation, oxidative stress, and Wnt ligand secretion. This negative regulation prevents hepatocyte proliferation and damage, ultimately inhibiting the development of HCC [16]. The immunosuppressive effect of TREM2 may lead to different outcomes at different stages of HCC. In the early stages, TREM2 limits inflammatory injury and tumor initiation [16]. However, in later stages, it can suppress antitumor immune responses and promote cancer progression [32]. This dual effect makes TREM2^+^ macrophages a double-edged sword in HCC development. Strategies that aim to activate TREM2 may restrict inflammatory injury and tumorigenesis in early stages but will suppress the antitumor immune response and drive cancer progression in later stages. A detailed evaluation of the potential side effects of therapeutically targeting TREM2 is warranted.

Our previous research indicated that immunosuppressive TREM2^+^ macrophages display a higher score for alternative M2 polarization than for classical M1 polarization. However, the in vitro M1/M2 dualistic model was unable to fully distinguish *TREM2*^+^ macrophages from *C1Q*^+^ macrophages [14]. Our analysis of cytokines indicated that BMDMs lacking *Trem2* secreted elevated levels of TNF-α but reduced levels of IL-1β. These findings suggest that macrophages in the tumor microenvironment exhibit more complex phenotypes, which aligns with conclusions from a prior study [34]. Through IHC analysis of mouse liver tumors, we found that *Trem2* deficiency did not significantly affect the number of intratumoral CD4^+^ and FOXP3^+^ cells. However, it did result in a significant increase in the number of intratumoral CD8^+^ T cells. Transcriptome profiling and cytokine array revealed increased secretion of chemokines, such as CXCL9 and CXCL10, by *Trem2*-knockout BMDMs. These findings align with a recent study showing that TREM2^+^ macrophages secrete lower levels of CXCL9 but increased levels of galectin-1, which mediates PD-L1 overexpression in vascular endothelial cells and impedes CD8^+^ T cell recruitment after transarterial chemoembolization in HCC [32]. In addition, TREM2^+^ macrophages diminish the recruitment and cytolytic activity of NK cells by disabling IL-18 signaling in lung cancer [35]. Conversely, TREM2, which is negatively correlated with immunosuppressive myeloid and T cell exhaustion signatures, plays an immunoprotective role during glioblastoma progression [36]. Moreover, myeloid TREM2 promotes MHCII-associated CD4^+^ T cell responses against gliomas [37]. Given that TREM2 has distinct expression patterns and various functions in tumor progression, the mechanism by which the expression of TREM2 affects the immune microenvironment merits further investigation.

Recent research has demonstrated that TGF-β signaling may promote TREM2^+^ NAM induction and that TGF-β1 can drive the differentiation of human monocytes to scar-associated macrophages (SAMs) expressing TREM2 [38]. In our investigation, we observed a significant positive correlation between TGF-β1 and TREM2 in clinical HCC samples. We further explored this relationship by adding exogenous TGF-β1, which increased mRNA and protein levels of TREM2 in human THP-1 cells and mouse BMDMs. Notably, the increase in TREM2 was reversed by a small-molecule TGF-β inhibitor, confirming the role of TGF-β1 in the induction of TREM2^+^ macrophages.

Metabolic reprogramming is a crucial factor in HCC development, and the activation of glycolysis is a common feature in various cancers. In accordance with the results of hallmark functional enrichment analysis, we hypothesized that TREM2^+^ macrophages enhance the malignant characteristics of HCC by promoting glycolysis. Furthermore, co-culture experiments revealed that inhibiting glycolysis with 2-DG eliminated differences in the proliferation of HCC cells between the WT and *Trem2* knockout groups. These findings underscore the importance of glycolysis in the crosstalk between TREM2^+^ macrophages and HCC cells. Previous studies utilizing single-cell RNA sequencing have also suggested that TREM2^+^ macrophages may drive HCC progression by modulating glycolysis [14], which aligns with the findings of this research. Using data from TCGA-LIHC dataset and in vitro studies, PKM2 emerged as a pivotal gene through which TREM2^+^ macrophages influence the glycolysis pathway in HCC cells. IL-1β was further identified as a protein that potentially increases PKM2 levels in HCC cells. Blocking the IL-1β receptor in liver cancer cells resulted in the disappearance of differences in PKM2 expression between the two groups, and there were similar changes in glucose consumption and lactate production, which are critical indicators of glycolytic function. IL-1β is only expressed by a small number of cell types, including macrophages, and has been identified as a promoter of glycolysis in multiple cancer types [39]. Although IL-1β is recognized as an inflammatory factor that has a tumor-suppressive role, investigations have indicated that tumor-associated macrophages with an M2 phenotype stimulate HCC cells to increase HIF-1α levels by releasing IL-1β, thereby promoting the epithelial‒mesenchymal transition and metastasis of HCC [40]. Studies on pancreatic cancer have demonstrated that IL-1β secretion by macrophages within the tumor microenvironment can induce angiogenesis and consequently promote cancer progression [41].

In summary, TREM2^+^ macrophages induced by TGF-β1 play a driving role in HCC progression by suppressing CD8^+^ T cells and promoting tumor cell glycolysis (Fig. 6H). Targeting TREM2^+^ macrophages could represent a therapeutic strategy for patients with HCC.

## Electronic supplementary material

Below is the link to the electronic supplementary material.

Supplementary Material 1

## Data Availability

Data are available from the corresponding authors upon reasonable request.
