# Supplementary material for: TREM2 promotes the formation of a tumor-supportive microenvironment in hepatocellular carcinoma
Source: J Exp Clin Cancer Res. 2025 Jan 21;44:20. doi: 10.1186/s13046-025-03287-w (PMC11748316; doi:10.1186/s13046-025-03287-w)
Supplement: Supplementary file 1 — Supplementary Material 1 [file 13046_2025_3287_MOESM1_ESM.docx]

**
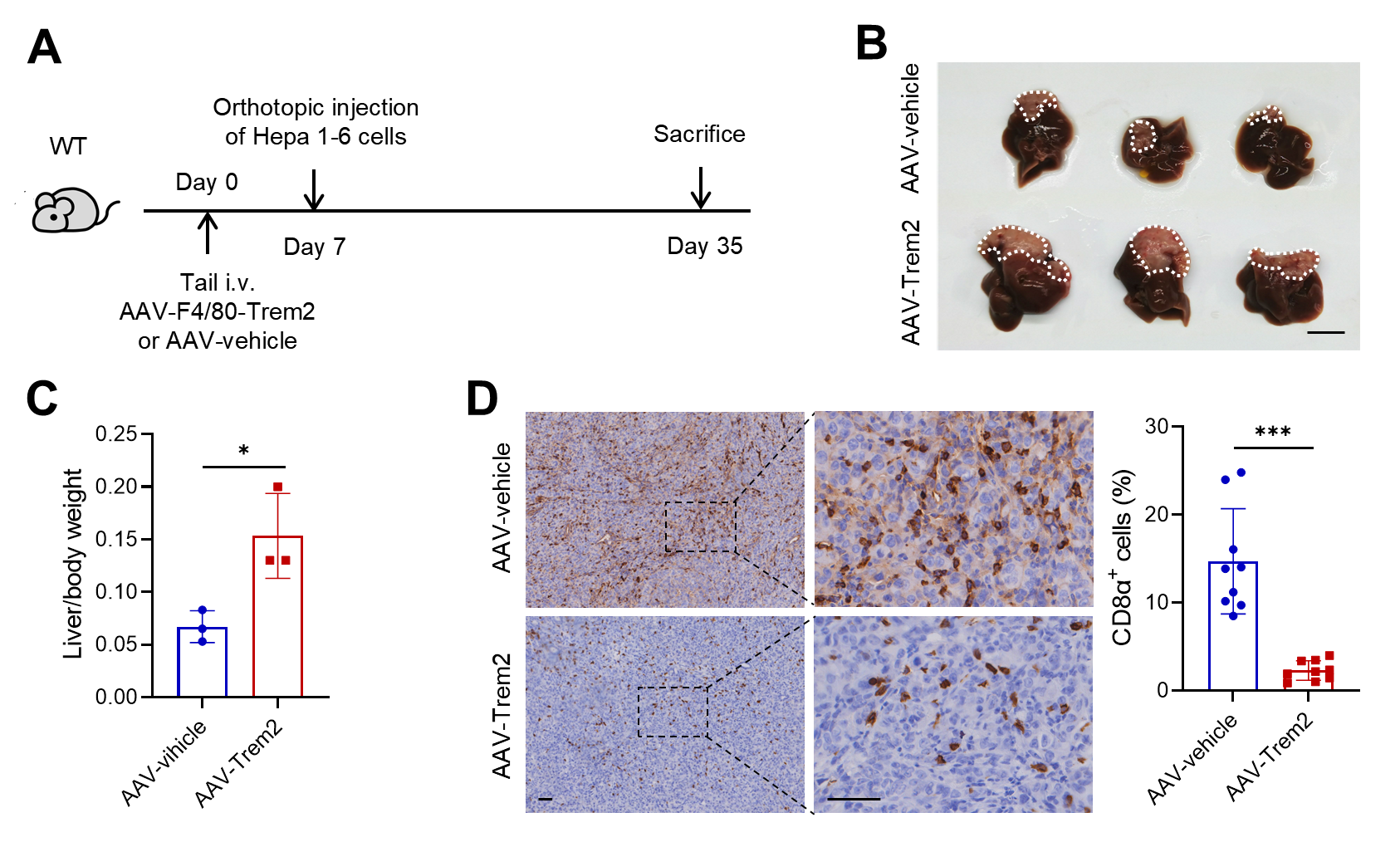
Supplementary Figure 1. TREM2 overexpression suppresses CD8^+^ T cell infiltration.** (A) Schematic procedure of Trem2 overexpression in liver macrophages. C57BL/6 mice were intravenously (i.v.) injected with Trem2-expressing adeno-associated viruses (AAV8-F4/80-Trem2, AAV-Trem2) or AAV-vehicle. After 7 days, the mice were orthotopically injected with Hepa 1-6 cells and sacrificed 35 days after AAV injection. (B) Livers of the orthotopic HCC model (n = 3). Scale bar, 1 cm. (C) Statistical analysis of the mouse liver/body weight ratio. (D) Representative images of IHC staining for CD8α in liver cancer tissues and corresponding statistics indicating the percentage of positively stained cells (n = 9 tumor areas from three mice). Scale bar, 50 μm. ns, not statistically significant; **P*<0.05, *** *P*<0.001.

**
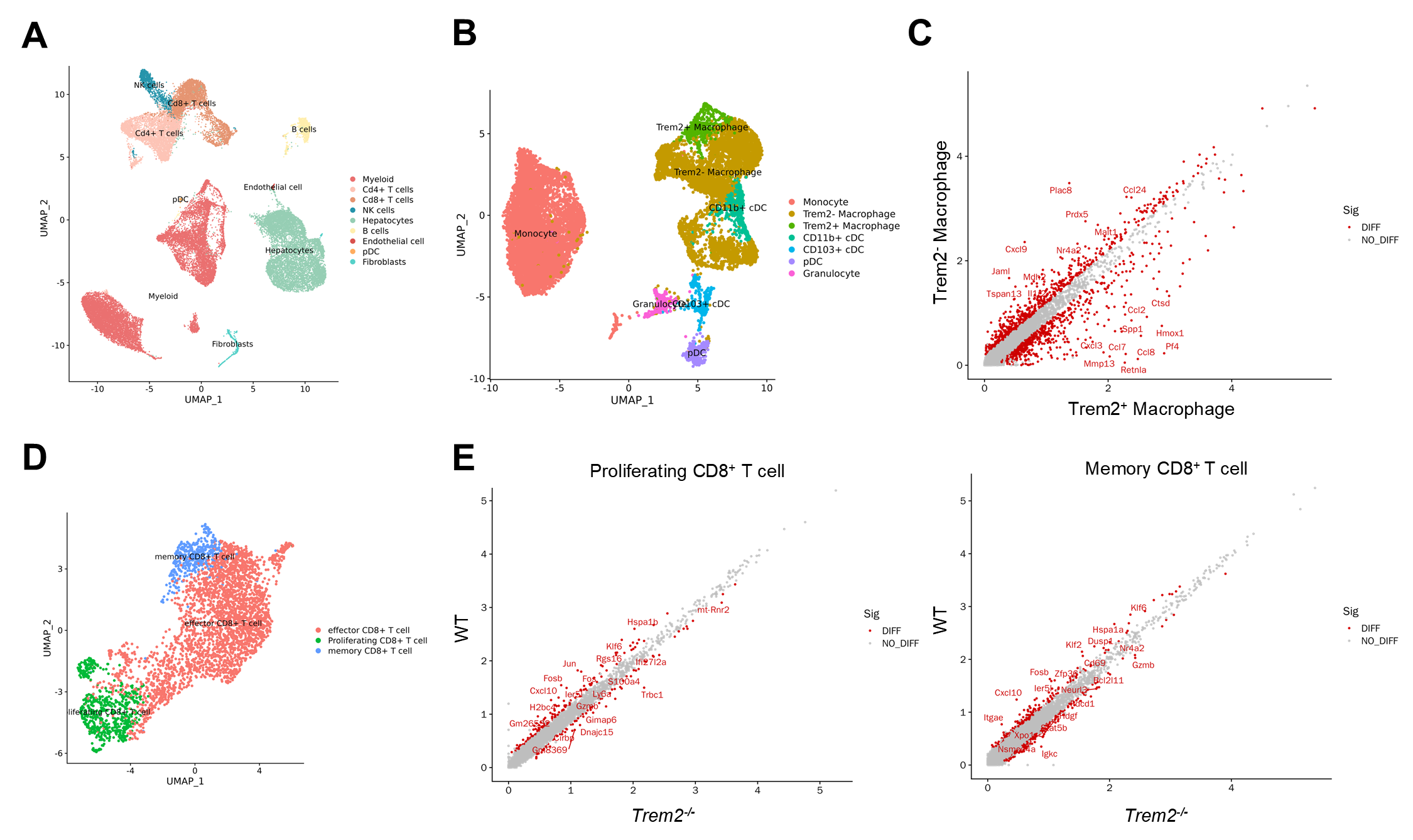
Supplementary Figure 2. TREM2^-^ macrophages increase Gzmb expression in CD8^+^ T cells.** WT C57BL/6 male mice received intrahepatic injections of 4×10^6^ Hepa 1-6 cells in combination with 1×10^6^ WT or *Trem2^-/-^* BMDMs. Liver tumors were harvested for single-cell sequencing (scRNA-seq; n = 2). (A) UMAP analysis of major cell types from the tumor tissue. (B) UMAP plot showing myeloid cell subclusters. (C) Differentially expressed genes between Trem2^+^ and Trem2^-^ macrophages. (D) UMAP plot showing secondary clusters of CD8^+^ T cells. (E) Differentially expressed genes in proliferating CD8^+^ T cells and memory CD8^+^ T cells between groups inoculated with WT and *Trem2^-/-^* BMDMs.

**
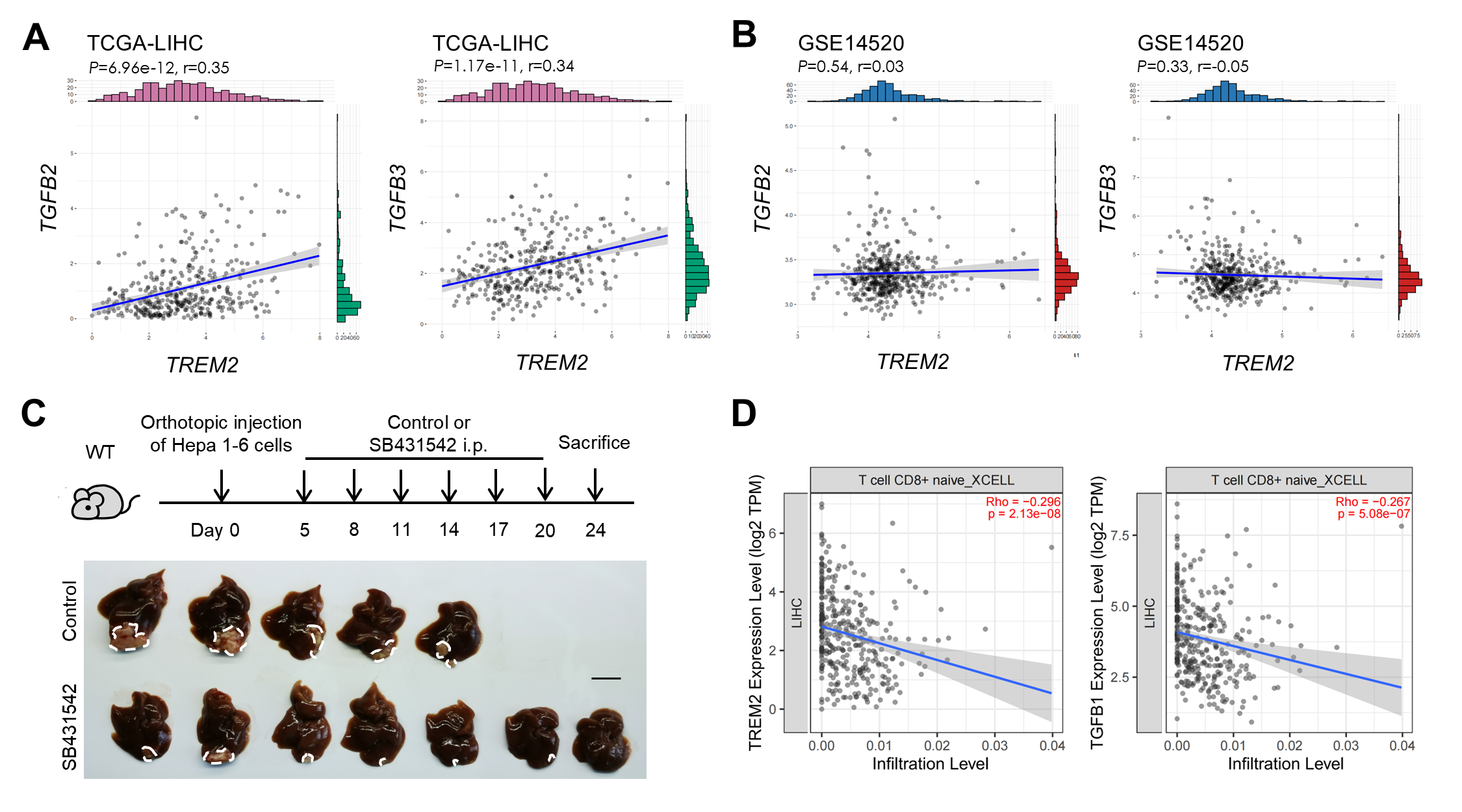
Supplementary Figure 3. TREM2 and TGFB1 are negatively correlated with CD8^+^ T-cell infiltration**. (A, B) Correlations of *TREM2* with *TGFB2* and *TGFB3* expression in TCGA-LIHC (A) and GSE14520 (B) cohorts. (C) In an orthotopic mouse model of HCC, the intraperitoneal (i.p.) injection of SB431542 resulted in a reduction in tumor size. Scale bar, 1 cm. (D) Correlations of *TREM2* and *TGFB1* expression with CD8^+^ naive T cell infiltration in TCGA-LIHC cohort. Analysis was performed via the TIMER webserver using the XCell algorithm. Spearman's correlation coefficients were calculated.

**
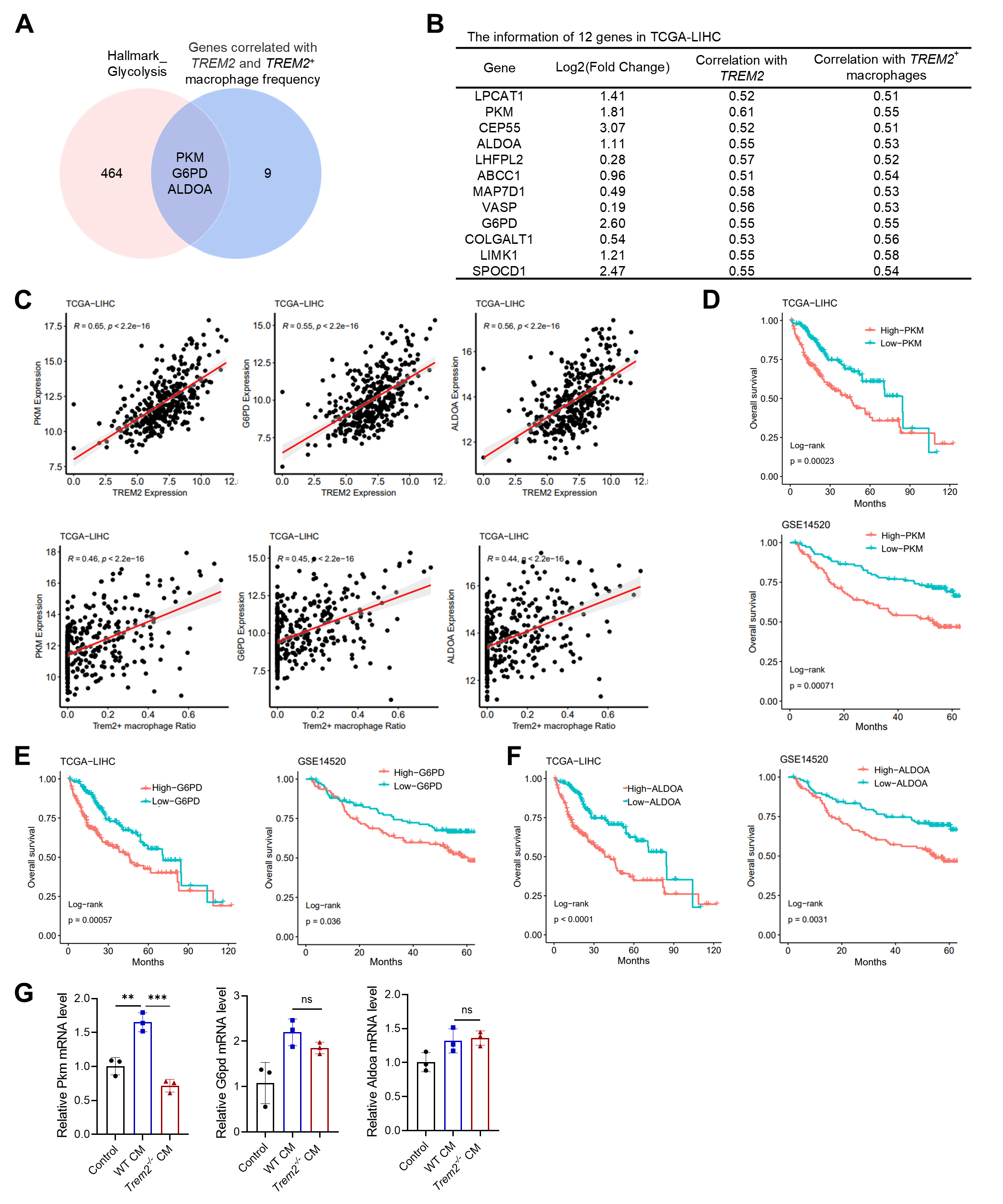
Supplementary Figure 4. TREM2 is associated with PKM expression in HCC.** (A) The intersection of genes in the Hallmark_Glycolysis gene set and genes correlated with *TREM2* expression and the percentage of TREM2^+^ macrophages estimated via CIBERSORTx [28]. (B) The correlation coefficients of genes related to *TREM2* expression and the percentage of TREM2^+^ macrophages. (C) Correlations of *PKM*, *G6PD*, and *ALDOA* with *TREM2* expression and the percentage of TREM2^+^ macrophages. (D‒F) Relationships between *PKM* (D), *G6PD*, (E), and *ALDOA* (F) expression and prognosis in TCGA-LIHC (left) and GSE14520 (right) cohorts. (G) mRNA expression of *Pkm*, *G6pd*, and *Aldoa* in Hepa 1-6 cells treated with the indicated CM types. ns, not statistically significant; ** *P*<0.01, ****P*<0.001.

**
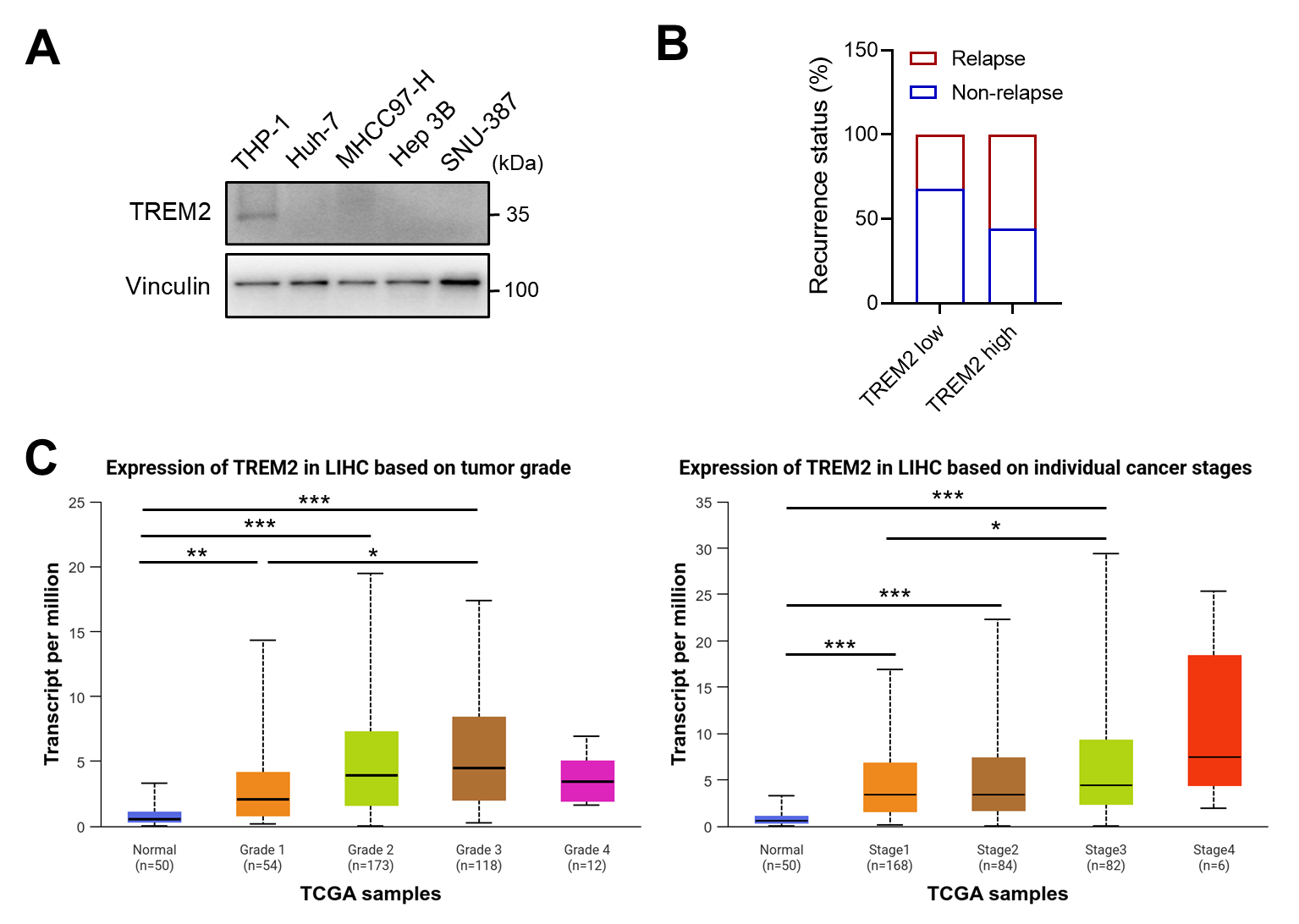
Supplementary Figure 5. TREM2 is highly expressed in macrophages and is correlated with a poor prognosis.** (A) Representative blots of TREM2 levels in THP-1 and HCC cells assessed by western blotting. Vinculin was used as the internal control. (B) Recurrence status in our cohort of HCC patients divided into high- and low-TREM2 groups, with the median IHC score used as the cutoff. (C) *TREM2* expression in TCGA-LIHC dataset based on tumor grade and cancer stage, sourced from the UALCAN web portal (<https://ualcan.path.uab.edu/analysis.html>).

**Supplementary Table 1. Primer sequences.**

| Symbol | Forward (5'→3') | Reverse (5'→3') |
| --- | --- | --- |
| *TREM2* | CCAGCCTGCATACTTGCCA | GGCAGAGTAGTCTCTTGCCAG |
| *ACTB* | CATGTACGTTGCTATCCAGGC | CTCCTTAATGTCACGCACGAT |
| *Trem2* | CTGGAACCGTCACCATCACTC | CGAAACTCGATGACTCCTCGG |
| *Pkm* | CGCCTGGACATTGACTCTG | GAAATTCAGCCGAGCCACATT |
| *G6pd* | CACAGTGGACGACATCCGAAA | AGCTACATAGGAATTACGGGCAA |
| *Aldoa* | TCGCTCCTTAGTCCTTTCGC | GGTGGCAGTGCTTTCCTTTC |
| *Actb* | GGCTATGCTCTCCCTCACG | GAGCAACATAGCACAGCTTCTCTTT |

**Supplementary Table 2. List of curated proteins regulating glycolysis.**

| Symbol |
| --- |
| CCL1, CCL20, CCL8, CXCL12, CXCL5, EGFR, FLT4, FSTL1, HGF, IL1B, IL6, LAMC1, ORM1, TFRC, VEGFA, WNT1, WNT10A, WNT10B, WNT11, WNT16, WNT2, WNT2B, WNT3, WNT3A, WNT4, WNT5A, WNT5A-AS1, WNT5B, WNT6, WNT7A, WNT8B, WNT9A, WNT9B |
